# A message from the new Editor-in-Chief

**DOI:** 10.1038/sj.bjc.6690110

**Published:** 1999-09-24


*British Journal of Cancer* (1999) **79**(1), 1
© 1999 Cancer Research Campaign

### A message from the new Editor-in-Chief

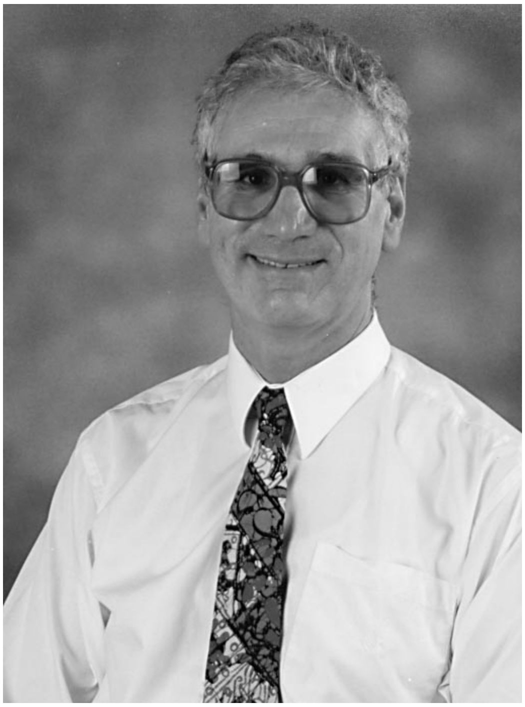

I feel honoured to take over as Editor-in-Chief of the *British Journal of Cancer*. Following the untimely death last June of Ged Adams (see obituary *Br J Cancer* **78**: 281), Peter Twentyman kindly stepped into the breach to act as editor until a long-term appointment could be made. Peter had been Editor-in-Chief prior to Ged, and it will be difficult to match the dedication of these two fine colleagues and friends.

The *British Journal of Cancer* will continue to serve the research community across a broad swathe of exciting developments in clinical and experimental cancer research. Although based in the UK as the clinical and scientific journal of the Cancer Research Campaign, the journal welcomes contributions worldwide, as can be seen from the contents of this issue. The importance and significance of the work it publishes is reflected in its international subscriptions and a readership predominantly outside the UK. The editorial team are discussing how the subjects within cancer research might be better classified, and we shall continue to present and review whatever is important in research on the understanding, diagnosis, treatment and prevention of cancer.

My own field of study is viral oncology and cancers related to AIDS. Oncogenic viruses have given us deep insight into molecular carcinogenesis; the discovery of oncogenes and p53 protein came through the study of viruses, for instance. Moreover, some 17% of human cancer is attributable to infections especially in developing countries, which offers potential for prevention through vaccination and screening. However, I can assure you that the *British Journal of Cancer* is not about to become a journal of infectious disease! We shall publish the best of all cancer research.

*Robin A Weiss*
*University College London*

